# P-2069. Outbreak of Shigella flexneri among people experiencing homelessness in Bernalillo County, NM 2021-2023

**DOI:** 10.1093/ofid/ofaf695.2233

**Published:** 2026-01-11

**Authors:** Henry Clay Carter, Angelica Solis, Leonard Noronha

**Affiliations:** University of New Mexico School of Medicine, Albuquerque, NM; University of New Mexico School of Medicine, Albuquerque, NM; University of New Mexico School of Medicine, Albuquerque, NM

## Abstract

**Background:**

Shigellosis is a diarrheal illness caused by gram-negative bacteria of the Shigella genus. It is transmitted via the fecal-oral route and associated with poor sanitation. In developed countries, incidence of shigellosis is low, with small outbreaks historically reported among childcare centers, prisons, and MSM communities. In the last 10 years a new pattern has emerged, with 9 outbreaks among people experiencing homelessness (PEH) in western cities of North America. Here we describe the demographic, clinical, and microbiological features of an outbreak that disproportionately affected PEH.

**Figure 1:**
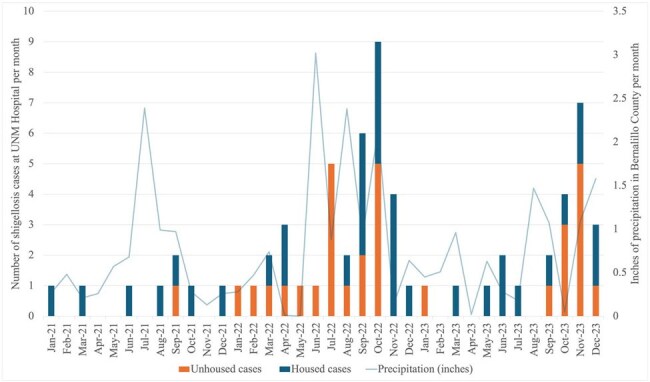
Timeline of shigellosis outbreak by housing status, with monthly precipitation in Bernalillo County superimposed

**Table 1:**
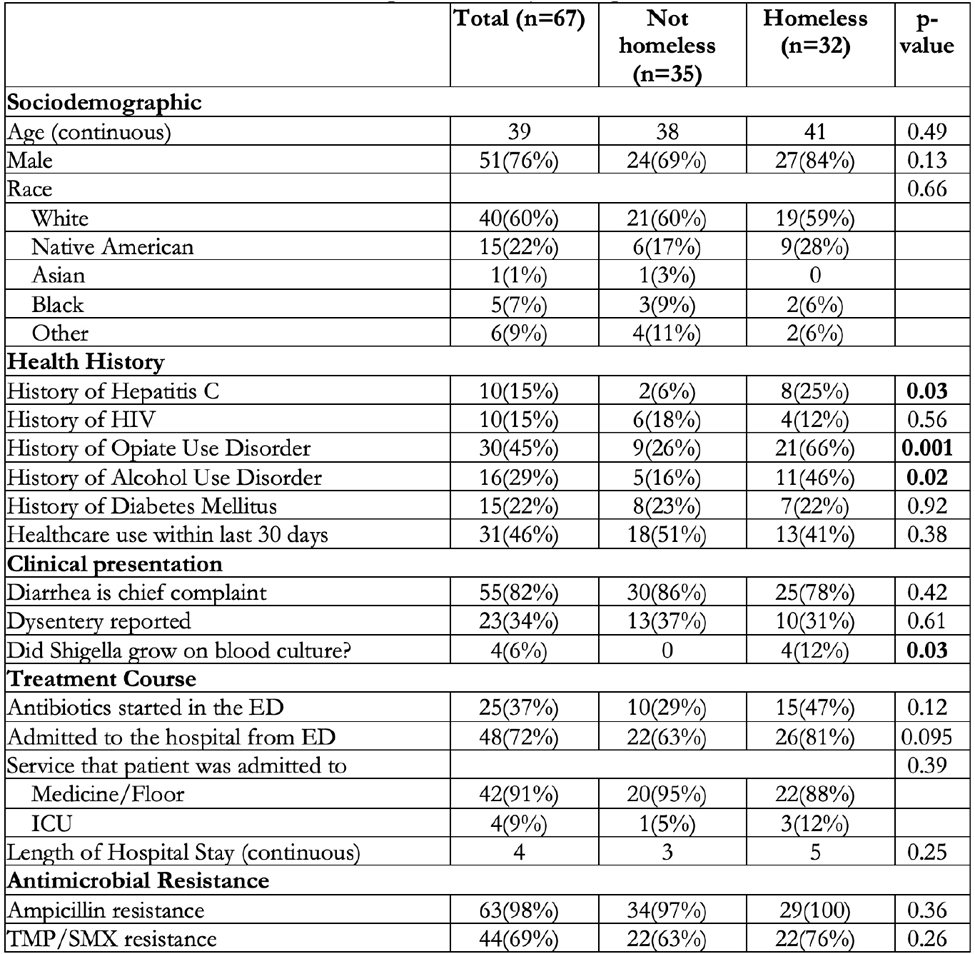
Characteristics of 67 UNM shigellosis cases by housing status

**Methods:**

This is a retrospective chart review of adults who presented to the UNM Hospital from 1/1/2021 through 12/31/2023 with stool culture positive for Shigella. A bivariable analysis compared risk factors and clinical courses between those who reported homelessness and those who did not.

**Results:**

We reviewed 67 cases of shigellosis, most presenting during two seasonal spikes in July 2022-November 2022 and September 2023-December 2023 that were preceded by high precipitation (Figure 1). Patients were mostly white (60%) and male (76%) with a median age of 39 (IQR 28, 52). Nearly half reported homelessness (48%). 34% presented with dysentery, 6% with bacteremia. Most required hospitalization (75%)[Table 1].

Patients reporting homelessness were significantly more likely to have history of hepatitis C (25%), opiate use disorder (66%), and alcohol use disorder (46%). PEH were significantly more likely to have bacteremia(12%).

All cases were caused by *Shigella flexneri.* Nearly all were resistant to ampicillin (98%) and most were resistant to TMP/SMX (69%). Fluoroquinolone resistance was not tested.

**Conclusion:**

Homelessness is an emerging risk factor for shigellosis. Our findings support the hypothesis that outbreaks among PEH are precipitated by inclement weather likely via overcrowding of shelters or encampments.

PEH with shigellosis have a higher burden of comorbidities. While PEH were more likely to present with bacteremia, their rates of hospitalization, ICU admission, and length of stay were not significantly different than housed patients.

While *Shigella sonnei* was the predominant or exclusive species in all prior outbreaks among PEH, all cases here were attributed to *S. flexneri.*

**Disclosures:**

All Authors: No reported disclosures

